# Probing the Structure of Toxic Amyloid-β Oligomers with Electron Spin Resonance and Molecular Modeling

**DOI:** 10.1021/acschemneuro.0c00714

**Published:** 2021-03-16

**Authors:** Martina Banchelli, Roberta Cascella, Cristiano D’Andrea, Giovanni La Penna, Mai Suan Li, Fabrizio Machetti, Paolo Matteini, Silvia Pizzanelli

**Affiliations:** † National Research Council of Italy, Institute of Applied Physics “Nello Carrara”, Sesto Fiorentino, I-50019 FI, Italy; ‡ University of Florence, Department of Experimental and Clinical Biomedical Sciences, I-50134 Firenze, Italy; § National Research Council of Italy (CNR), Institute of Chemistry of Organometallic Compounds (ICCOM), Sesto Fiorentino, I-50019 FI, Italy; ∥ National Institute for Nuclear Physics (INFN), Section of Roma-Tor Vergata, I-00133 Roma, Italy; ⊥ Institute of Physics, Polish Academy of Sciences, Al. Lotnikow 32/46, 02-668 Warsaw, Poland; # Institute for Computational Science and Technology, 6 Quarter, Linh Trung Ward, Thu Duc District, 700000 Ho Chi Minh City, Vietnam; ∇ University of Florence, Department of Chemistry “Ugo Schiff”, Sesto Fiorentino, I-50019 FI, Italy; ○ National Research Council of Italy (CNR), Institute of Chemistry of Organometallic Compounds (ICCOM), I-56124 Pisa, Italy

**Keywords:** Alzheimer’s disease, amyloid-β (Aβ) peptides, Cu^2+^, double electron−electron resonance (DEER)

## Abstract

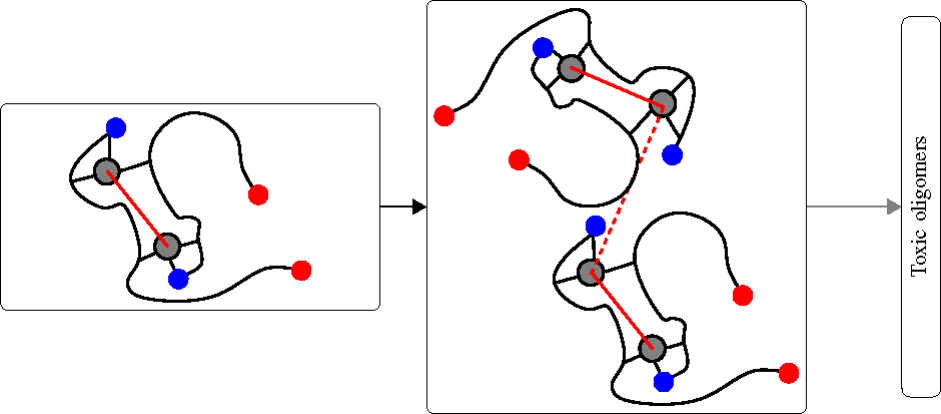

Structural models of the toxic species involved in the development of Alzheimer’s disease are of utmost importance to understand the molecular mechanism and to describe early biomarkers of the disease. Among toxic species, soluble oligomers of amyloid-β (Aβ) peptides are particularly important, because they are responsible for spreading cell damages over brain regions, thus rapidly impairing brain functions. In this work we obtain structural information on a carefully prepared Aβ(1-42) sample, representing a toxic state for cell cultures, by combining electron spin resonance spectroscopy and computational models. We exploited the binding of Cu^2+^ to Aβ(1-42) and used copper as a probe for estimating Cu–Cu distances in the oligomers by applying double electron–electron resonance (DEER) pulse sequence. The DEER trace of this sample displays a unique feature that fits well with structural models of oligomers formed by Cu-cross-linked peptide dimers. Because Cu is bound to the Aβ(1-42) N-terminus, for the first time structural constraints that are missing in reported studies are provided at physiological conditions for the Aβ N-termini. These constraints suggest the Aβ(1-42) dimer as the building block of soluble oligomers, thus changing the scenario for any kinetic model of Aβ(1-42) aggregation.

## Introduction

Alzheimer’s Disease (AD) is the most representative form of dementia in humans. The disease is associated with cognitive degradation caused by neuron death. The most investigated molecular event associated with cell death is the aggregation of amyloid-β (Aβ, hereafter) peptides to form extracellular fibrils.^1−6^ Aβ peptides are the byproduct of amyloid precursor protein (APP).^7^ The relative abundance of amyloid peptides in both normal subjects and AD patients is still debated.^8,9^ Recent analyses reported the ranking of abundance as Aβ(4-42) ∼ Aβ(1-42) > Aβ(1-40), with strong indications of Aβ(4-42) peptide as the most abundant in AD patients (see ref (10) and discussion therein). Most of the *in vitro* studies, however, are still concentrated on Aβ(1-42).

The short-living monomeric form of any Aβ species is an intrinsically disordered protein, as definitely shown by nuclear magnetic resonance (NMR)^11^ and Förster resonance energy transfer (FRET)^12^ experiments. Monomers rapidly form relatively soluble oligomers, then highly ordered insoluble protofibrils, and eventually extended fibrils and plaques.^13,14^ Soluble oligomers are toxic,^2,15−21^ because they induce membrane disorder and pores,^22,23^ affect membrane lipid order and permeability,^24,25^ inhibit hippocampal long-term potentiation,^2^ induce τ hyperphosphorilation and cytoskeleton changes,^26^ and interact with cell receptors.^27,28^ The Aβ dimers already show neurotoxicity.^29−31^ The characterization of Aβ oligomers is crucial to understand their role as diffusible toxic species before the late aggregation process occurs.^32−36^ Aβ oligomers are, therefore, early biomarkers of AD.

Metal ions such as zinc, copper, and iron are found at high concentrations in fibrils and plaques extracted from the AD-affected area of the brain.^37−41^ Metal ions interact with APP and Aβ, and complexes between metal ions and Aβ are potentially important species before the aggregation of Aβ occurs. In addition to its biological relevance, the affinity of magnetic ions, like Cu^2+^, for Aβ peptides can be used to probe structural features characteristic of the oligomerization state of Aβ.

In this work we combined extended molecular models^42^ that have been used to explain ion mobility mass spectrometry (IM-MS) data^43^ and oxidative pathways,^44^ with advanced electron spin resonance (ESR) techniques, like double electron–electron resonance (DEER), to estimate Cu–Cu distance distribution in Cu–Aβ(1-42) oligomers. Copper ions are used as ancillary magnetic probes to address oligomer structure.

The 1:1 Cu–Aβ(1-42) complex is suitable to DEER measurements irrespective of the aggregation state. The possibility to observe changes in DEER measurement with incubation time, concentration, and other conditions is extremely important to monitor the change of Cu–Cu distance with the aggregation state. Soluble compact oligomers are observed by IM-MS as species coexisting with protofibrils and fibrils. Indeed, according to computational models consistent with the IM-MS data of soluble oligomers, the minimal distance between Cu^2+^ ions in compact dimers and tetramers is around 2 nm. The shape of the Cu–Cu distance distribution can in theory discriminate between globular and shaped oligomers, the latter behaving as template protofibrils and inducing the formation of elongated fibrils. Globular toxic oligomers are supposed to be off-pathway to elongated protofibrils, and they are more suitable to diffuse in the cerebrospinal fluid compared to larger aggregates, thus providing a mechanism for the propagation of toxicity in the central nervous system.

Recently, a reproducible protocol for isolating toxic globular oligomers^45^ has been applied to spectroscopic investigations.^46^ This study shows that by properly tuning sample preparation, specific properties of samples that represent the AD toxic agents can be investigated. The oligomers representative of toxic state are indicated as A+. Surface enhanced Raman spectroscopy (SERS) allows a characterization of this toxic state, correlated with atomic force microscopy (AFM) approximate measurements of oligomers’ sizes. In this work, we report DEER experiments probing Cu^2+^ centers. Experiments are performed on Aβ samples prepared as in previous studies, apart from the addition of Cu^2+^ in conditions that do not perturb the morphology of the A+ toxic state.

While electron spin echo envelope modulation (ESEEM) measurements have been reported for Cu–Aβ models,^47^ the DEER experiment has been performed only on different systems of biological interest.^48,49^ The Cu complex of the prion N-terminus (octarepeat) binds, in humans, up to 4 Cu^2+^ ions. Each of the 4 N-terminal repeats PHGGGWGQ binds one Cu ion. This system has been widely investigated with ESEEM experiments,^50^ and only recently, DEER experiments were performed on prion complexes.^51^ The Cu,Zn-superoxidedismutase (Cu,Zn-SOD) enzyme has also been investigated, because it is dimeric in water solution at physiological conditions.^52^ Therefore, the present work describes, for the first time, a DEER experiment for Cu–Aβ(1-42) oligomers.

## Results and Discussion

According to our previous work,^46^ where the conditions of oligomer formation were tightly controlled, we prepared two samples displaying different aggregation pathways: sample **1**, producing cytotoxic oligomers^45^ (indicated as A+) and slowly evolving into less toxic amorphous aggregates (indicated as A−); sample **2**, constituted by protofibrils slowly evolving into mature fibrils. In the former sample, Cu–Aβ(1-42) concentration is 25 μM, whereas in the latter, it is 75 μM. The preparation of the samples traces that reported in ref (46), and it is described, along with the techniques used in this work, in the Materials and Methods. In this work the procedure adopted in order to add Cu^2+^ ions to the peptide minimizes the perturbation of the peptide behavior (see Materials and Methods).

**Figure 1:**
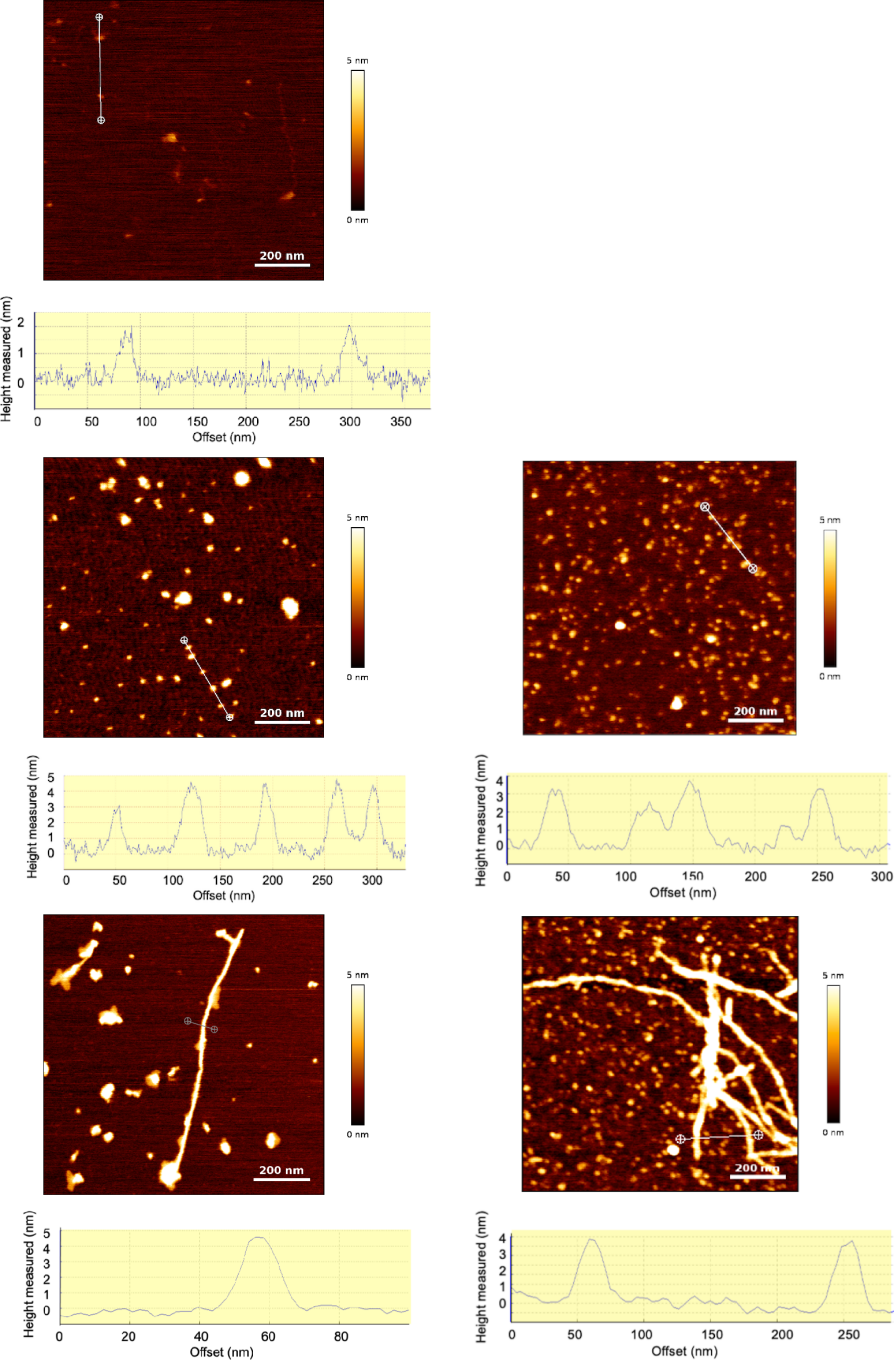
AFM images and cross section profiles (graphs) along the lines drawn on the corresponding images: (a) PBS solution only; (b) Aβ(1-42) oligomers (A+) with Cu^2+^ (sample **1**, 25 μM 1:1 Aβ(1-42)–Cu molar ratio), (c) same sample with no Cu; (d) Aβ(1-42) fibrils with Cu^2+^ (sample **2**, 75 μM 1:1 Aβ(1-42)–Cu molar ratio), (e) same sample with no Cu.

The morphology of both samples (Figure 1b,d) is identical to that displayed by a Cu-free sample^46^ (Figure 1c,e). In particular, the cross section profiles reveal height values of 4.4 ± 2.4 nm for the oligomers, in agreement with the values observed in the Cu-free samples. Sample **2** contains small fibrils together with small irregular particles (Figure 1d), a picture that is consistent with the protofibril stage where large fibrils are not yet the dominant aggregated form. In the absence of Cu, protofibrils are more clearly the dominant form (Figure 1e). The presence of the small irregular particles in sample **2** is in line with previous observations showing that the addition of Cu to Aβ(1-42) favors amorphous aggregates.^53,54^ The comparison between images of samples with and without Cu shows that the amount of AFM visible particles is lower with Cu than in the absence of Cu at the same incubation time. This is consistent with the low aggregation rate of monomers observed in the 1:1 Cu–Aβ(1-42) ratio compared to Cu-free Aβ.^53^ The presence of significant amounts of soluble monomers and oligomers is therefore expected for 1:1 Cu–Aβ(1-42) even at long incubation times (48 h).

**Figure 2:**
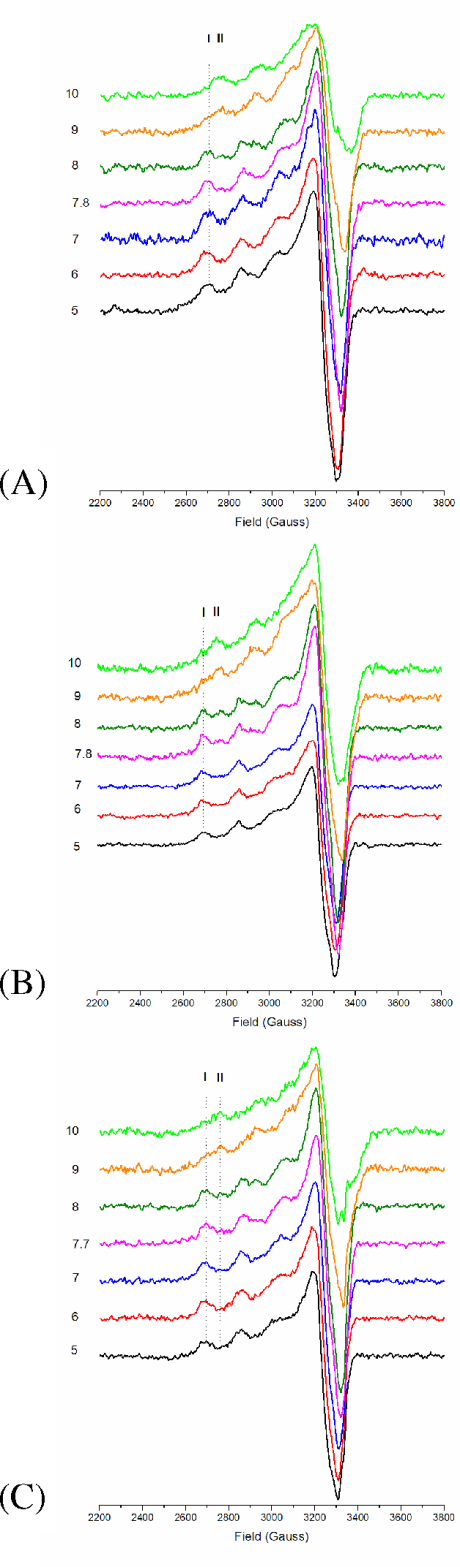
X-band CW ESR spectrum of sample **1** at different pH (different colors in each panel) and at different incubation times (different panels). (A) Time zero; (B) after 24 h; (C) after 48 h. Black pH = 5; red pH = 6; blue pH = 7; purple the pH of the sample before any pH shift (see Materials and Methods); dark green pH = 8; orange pH = 9; light green pH = 10. Panel A can be compared with Figure 1 in ref (55) for Aβ(1-28) 50 μM. The vertical lines indicate the leftmost parallel band of the two different species (components I and II) identified for Aβ(1-28).

In order to gain insight into copper coordination, the X-band continuous wave spectra of sample 1 were recorded along with incubation time. Because copper coordination depends on pH, the pH of sample **1** was also measured, and spectra at different pH values were collected (see Materials and Methods for details). These spectra are displayed in Figure 2, with panels A, B, and C showing those recorded at time zero and after 24 and 48 h of incubation, respectively. The spectra recorded at time zero represent the monomeric references. The pH measured on the original samples after the three incubation times is equal to 7.7–7.8. This value is slightly shifted compared to that expected for the used PBS buffer (7.4) and does not depend on the incubation time. It is due to the NaOH added before PBS addition in order to start with a monomeric state (see Materials and Methods).

The spectra recorded at this pH value after the three times are represented by the purple lines in Figure 2. They show that the copper coordination mode assumed in our model (indicated as I in Figure 2) dominates over II, which involves the deprotonation of the Ala 2 amide group. The coordination modes detected at different pH values are quite insensitive to the incubation time and reflect those observed for Cu–Aβ(1-28) at the same selected pH values in NEM buffer (Figure 1 of ref (55)) and for Cu–Aβ(1-16) in PBS (pH = 6.3, 6.9, 8.0; Figure 1, right panel of ref (56)). Both Aβ(1-16) and Aβ(1-28) peptides contain the Aβ(1-42) relevant Cu-binding ligand atoms in the disordered N-terminus. Compared to Aβ(1-42), the shorter peptides aggregate at a lower rate, and thus, the published ESR spectra display mainly the behavior of Cu–Aβ monomers. One should notice that, for Aβ(1-42), the transition between the two coordination modes, I and II, begins at a slightly lower pH compared to that of Aβ(1-28), in line with the trend observed in ref (55) with the Aβ peptide length, where the transition is observed at pH = 8.0 in Aβ(1-28) and 8.7 in Aβ(1-16).

We remark here that the change of Cu-binding atoms when ESR component II becomes dominant strongly affects oxidoreductive properties of Cu–Aβ,^57−59^ but there is no evidence about possible consequences in the cross-talk between N- and C-termini in the peptide.

In order to test the presence of Cu coordination sites regularly distributed in space within a distance ranging between 1 and 8 nm, we applied DEER to samples **1** and **2**.

**Figure 3:**
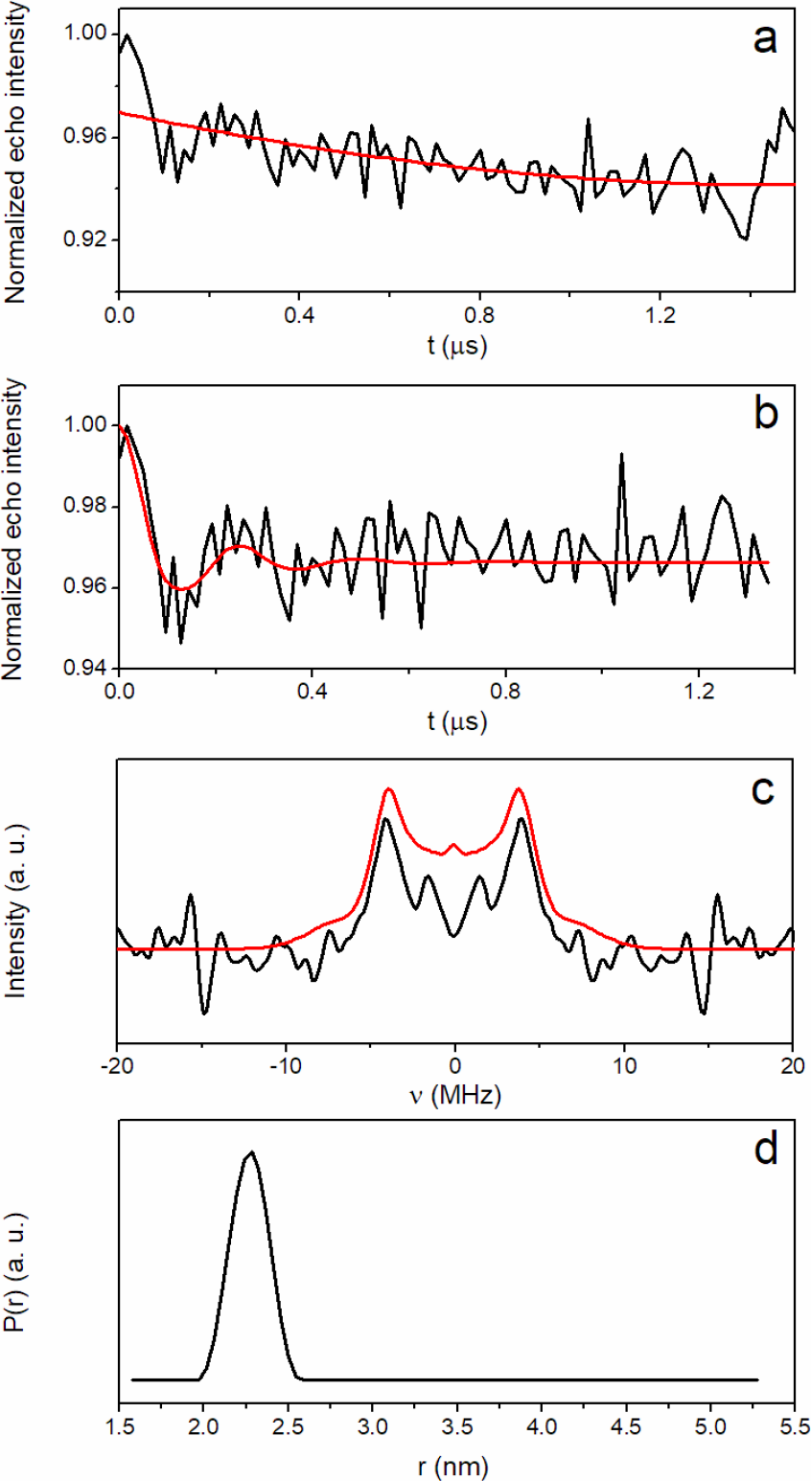
DEER measurement for sample **1** after 24 h of incubation. (a) DEER time trace (black line) and background fitting curve (red line). (b) Background corrected DEER time trace (black line) and best fitting curve (red line). (c) Frequency spectra corresponding to the time traces shown in part b. (d) Distance distribution. The analysis of the DEER time trace was conducted using DeerAnalysis2019 software,^93^ freely available at http://www.epr.ethz.ch/software/index.

**Figure 4:**
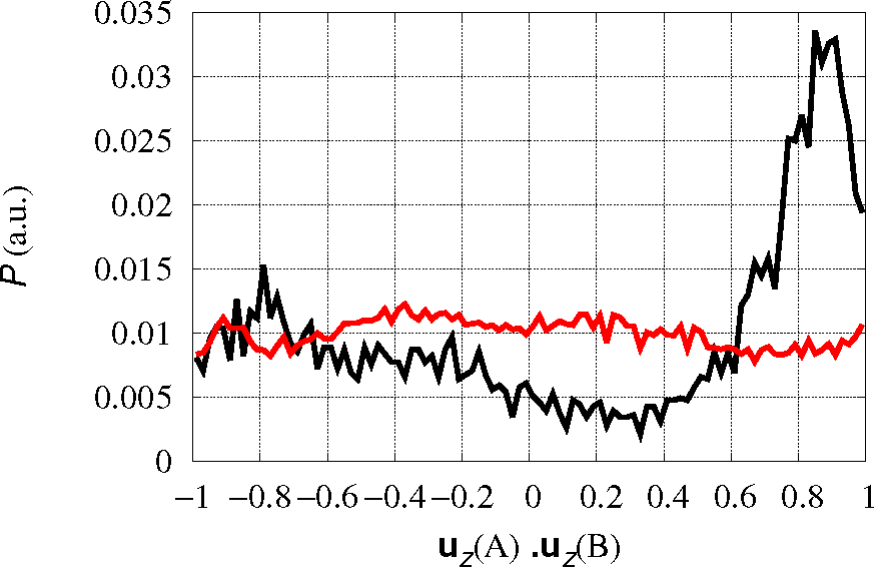
Distribution *P* of orientation of different Cu-coordination planes represented by the scalar product between the unit vectors normal to coordination plane (**u**_*z*_) in different monomers. The *z* direction is determined as that perpendicular to the plane formed in each configuration by Cu–Nδ(His 6) and Cu–Nϵ(His 13) bonds. The black curve is obtained averaging over tetramers formed by Cu-cross-linked dimers; AB and CD pairs are averaged. The red curve is obtained averaging over AC, AD, BC, and BD pairs in separated dimers, where Cu-coordination planes are not correlated.

The DEER time trace for sample **1** is shown in Figure 3a. It displays sufficiently pronounced oscillations, characteristic of a single dominant dipolar interaction within the optimal DEER distance range. Figure 3b shows the DEER time trace where the background is removed from the original data. The Fourier transform of the background corrected signal is reported in Figure 3c. The data were analyzed by using the model-free Tikhonov regularization method^60^ to obtain most probable distances. This method is acceptable if an error of 1–3 Å on most probable distances is tolerated and when orientational selectivity effects are weak.^61^ Unfortunately, the low signal-to-noise of the data and instrumental limitations prevented us from probing the significance of the orientational effects by collecting additional data sets. On the other hand, simulated DEER data show that orientational effects are substantially reduced by sufficiently large distributions of the relative orientations of the *g* tensors located on the two interacting Cu^2+^.^61,62^ Indeed, the atomistic models of tetramers reveal a broad distribution of the angle between the vectors normal to the Cu binding ligand planes for copper ions in the same dimer, as displayed in Figure 4. The normal vectors tend to be parallel with a quite large standard deviation, which is about 20°.

By applying this simplified analysis, the dominant frequency shift is attributed to a unimodal Cu–Cu distance distribution (Figure 3d), with a maximum corresponding to a distance of 2.2–2.3 nm.

In order to relate the distribution of Cu–Cu distance estimated by the DEER analysis with structural constraints for Aβ oligomers, we used the statistics of Cu–Aβ(1-42) tetramers (say ABCD, indicating the monomers with letters A–D) collected starting from the assembling of 2 × Cu–Aβ(1-42) (non-Cu-cross-linked) and [Cu–Aβ(1-42)]_2_ (Cu-cross-linked) preformed dimers^42,63^ (see Materials and Methods for details). A third model of 1:2 Cu–Aβ(1-42) tetramers 2 × [Cu–Aβ(1-42)–Aβ(1-42)] was also used for comparison. We are reminded that, in our models, the Cu binding to Aβ(1-42) is constrained to the dominant species contributing to ESR spectra at pH ∼ 7,^64−66^ which is component I shown in the ESR spectra described above. The binding is quite stable, being characterized by a dissociation constant *K*_d_ ranging from 10^–9^ M for the Aβ monomer^67,68^ to 10^–11^ M for aggregated forms.^69^ Recent measurements for Cu(II)–Aβ(1-16) show a significant dependence of *K*_d_ on pH and chain modifications;^70^ yet, they confirm a value of log *K*_d_ = −9.8 M for Cu–Aβ(1-16) (i.e., the Cu-binding region of Aβ) at pH = 7.4.

**Figure 5:**
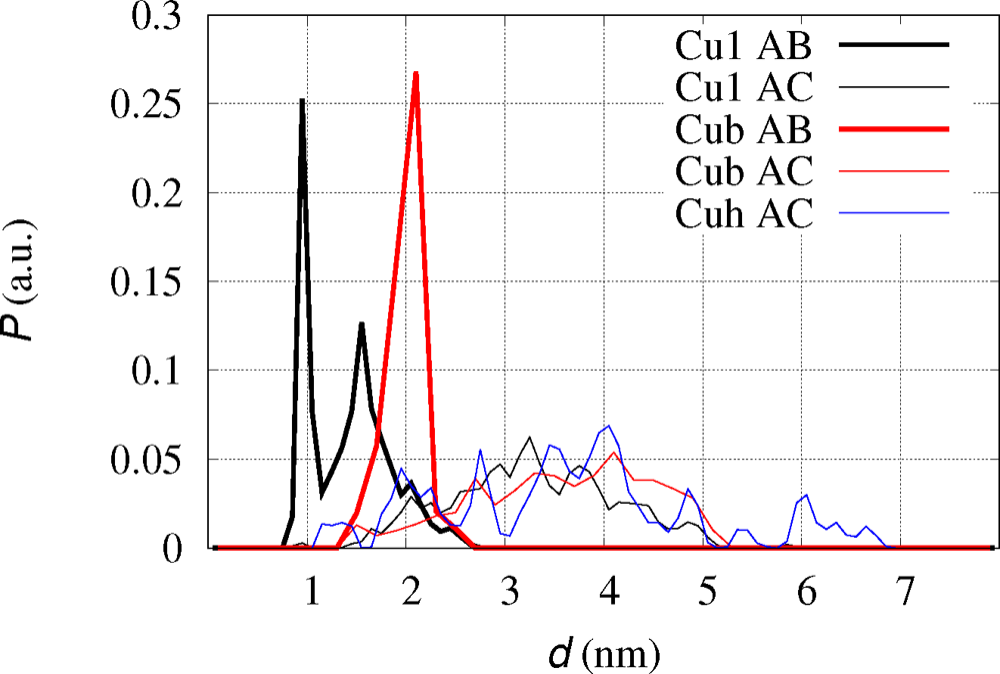
Distribution *P* of Cu–Cu distances (*d*) in simulated ABCD tetramers.^42^ Thick lines are Cu–Cu distances within AB and CD dimers: black line non-Cu-cross-linked dimers (Cu1 AB); red line Cu-cross-linked dimers (Cub AB). Thin lines are Cu–Cu distances between monomers not involved in preformed dimers: AC, AD, BC, BD pairs (4 samples) for Cub (black line); AC, AD, BC, BD pairs (4 samples) for Cu1 (red line); AC pairs (1 sample) for Cuh model (blue line). All the curves are normalized to give the same integral, despite the number of samples being different.

In Figure 5, the distribution of Cu–Cu distances obtained by these statistics is displayed. It can be noticed that the Cu1 model displays two peaks at distance of 1 and 1.6 nm, when the Cu ions belong to the same preformed dimer (indicated as AB in the figure, thick black line), while a single peak at 2.1 nm is displayed for the Cub model at the same conditions (red thick line). This shows that, when dimers are formed as Cu-cross-linked monomers, the Cu centers tend to be kept at larger distances than when the Cu ions are not involved in cross-links between two peptides. As for distances involving Cu centers not involved in preformed dimers (indicated as AC in the figure, thin lines), the distribution is broad in all models, though a small contribution of Cu1 and Cuh models is still visible at 1 nm. In model Cuh, only one monomer in each preformed dimer contains Cu; therefore, only contributions by AC pairs are computed. The distribution of Cu–Cu AC distance (blue thin line in Figure 5) is broader than Cu1 and Cub, displaying a larger number of assembled structures able to accommodate two separated Cu-binding sites in tetramers, when Cu-bound peptides are intercalated by Cu-free peptides. Average distance is for Cu1 1.7 ± 0.5 and 3.3 ± 0.8 nm for AB and AC pairs, respectively; for Cub 2.0 ± 0.2 and 3.6 ± 0.9 nm for AB and AC pairs, respectively; and for Cuh 3.6 ± 1.3 nm for AC pairs. Total average distance is 2.7 ± 1 and 3.0 ± 1 for Cu1 and Cub tetramers, respectively.

It is possible to determine the statistical difference between the two data sets related to AB dimers in tetramers (Figure 5, the two thick curves) and between those of AC dimers (thin curves). This difference is given by the *p*-value, where the tolerance for a significant difference is usually assumed when *p* < 0.05. The *p*-value is lower than 0.001 for thick curves, and it is 0.025 for thin curves, both in Cu1 and Cub models. Therefore, despite the large error affecting the average obtained by each distribution, distributions corresponding to black and red curves are statistically different, and on average, the Cu–Cu distance within non-Cu-cross-linked dimers is significantly shorter than that within Cu-cross-linked dimers.

The comparison between the distance dominating the dipolar interactions between Cu ions obtained in sample **1** and the molecular statistics for oligomers (so far tetramers) obtained by atomistic simulations shows that a Cu-cross-linked dimer assembled into larger oligomers is more consistent with the DEER data than non-Cu-cross-linked oligomers. The 1:1 non-Cu-cross-linked binding of Cu to Aβ produces configurations where preformed dimers approach one each other with Cu ions too close to be consistent with the DEER experiment. Cu–Cu distances where ions are not involved in an AB dimer are expected to contribute to the background.

The dominant distance indicated by the analysis of DEER data of sample **1** encompasses many possible structures of Cu–Aβ(1-42) oligomers. Possible structures displaying a Cu–Cu distance shorter than 1 nm or longer than 8 nm are transparent to the DEER technique. However, the detection of DEER signal corresponding to a Cu–Cu distance of ∼2 nm shows that not many of such structures occur.

The structures contributing to the ∼2 nm distance can be soluble dimers or structures with Cu pairs with regular Cu–Cu distance embedded in larger oligomers, like those displayed by AFM images. For instance, Cu-cross-linked soluble dimers can coexist with oligomers where only two Cu ions are embedded. However, the consistency between the mostly unimodal DEER distribution and the tetramer models built by assembling Cu-cross-linked dimers reveals that the topology of dimers can be easily preserved in tetramers. Inspired by this observation, we are exploiting the assembly of dimers into larger models of oligomers, up to dodecamers. This bias of pairing monomers in the assembling process has been suggested by early IM-MS measurements.^32^

Further models will also be useful to explore more intricate possible Cu-cross-linking, like the assembly of tetramers where monomers are Cu-cross-linked in annular topologies and Cu–Cu pairs display more than one regular distance in the 1–8 nm range. Indeed, the occurrence of more than one regular distance can not be fully excluded by the DEER measurement of sample **1** after 24 h of incubation, and further studies are required. We remark that the DEER signal of sample **1** after 24 h is the first affirmative DEER measurement for Cu–Aβ complexes, so far.

**Figure 6:**
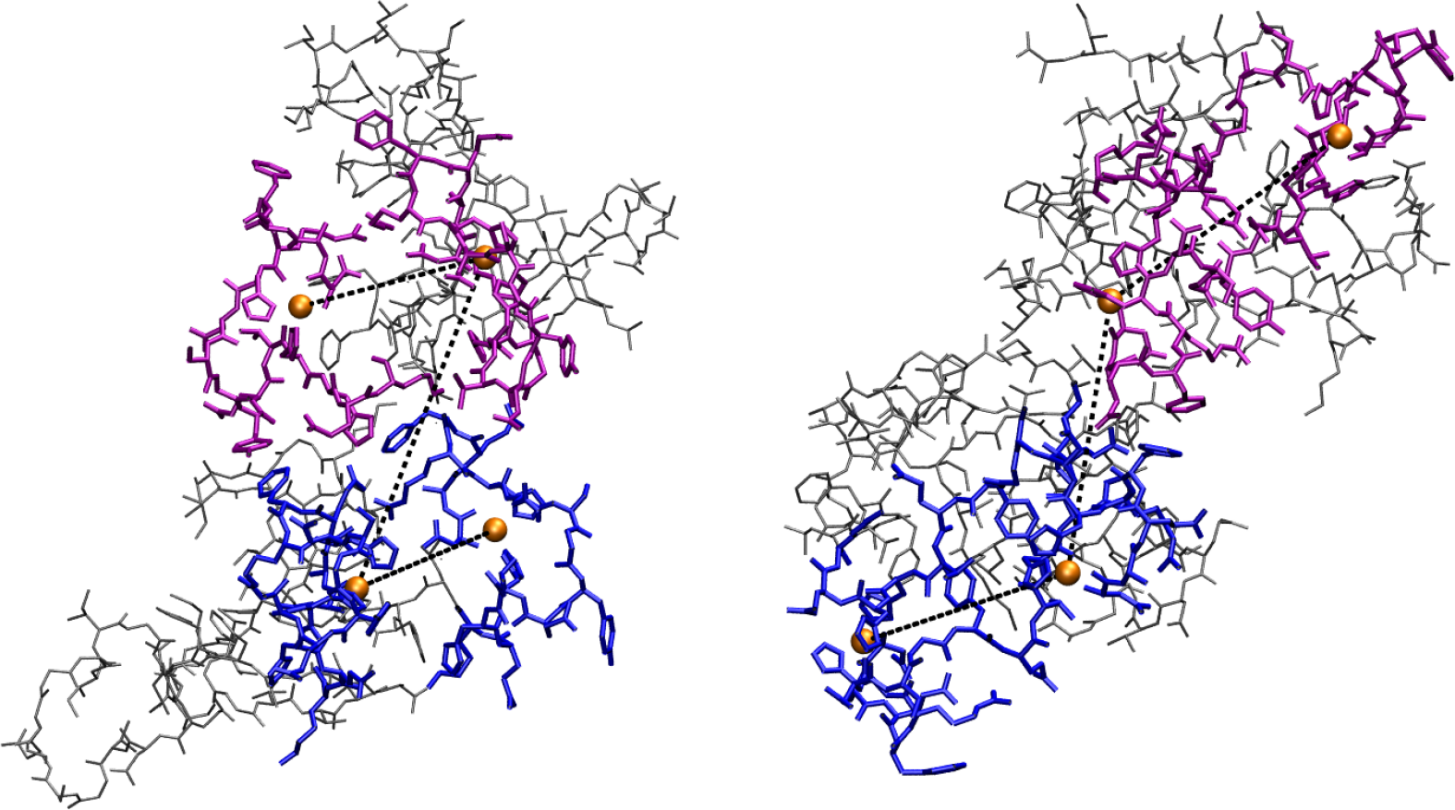
Representative configurations (see text for details) of simulated tetramers. (a) Tetramer formed by non-Cu-cross-linked dimers, *d*(Cu(A)–Cu(B)) = 1.6 nm, *R*_g_ = 2.0 nm, 1 individual. (b) Tetramer formed by Cu-cross-linked dimers, *d*(Cu(A)–Cu(B)) = 2.1 nm, *R*_g_ = 1.9 nm, 1 of 14 individuals. The N-termini (residues 1–16) of A and B monomers are in blue; those of C and D are in purple. All the other residues are in gray. Cu is the orange sphere. Atomic and bond radii are arbitrary. H atoms are not displayed. The VMD program^100^ is used for molecular drawing.

The constraint of Cu–Cu separation in each [Cu–Aβ(1-42)]_2_ cross-linked dimer is due to the separation of A and B N-termini because of the interaction between the N-terminus of peptide A and His 13 (or His 14) in peptide B, mediated by Cu. This constraint forces an antiparallel arrangement of N-termini of A and B monomers. The absence of Cu-cross-links allows for a parallel arrangement of N-termini that has the consequence of separating the C-termini of the two approaching dimers. Representative tetramer configurations (see Materials and Methods for details) contributing to the maximum of Cu(A)–Cu(B) distribution in Figure 5 (thick black and red lines) are displayed in Figure 6, parts a and b, respectively.

The structural constraint due to Cu-cross-links is also responsible for the approach of Tyr 10(A) to Tyr 10(B), as well as other residues sensitive to oxidation. Indeed, a short Tyr–Tyr distance is found, both in experiments^44^ and in simulations,^42^ more probable in Cu-cross-linked dimers (Cub model) than in non-Cu-cross-linked ones (Cu1 model).

As for sample **2**, no DEER time trace was detected. The absence of DEER time trace for sample **2**, that is, in a fibril state obtained in an environment identical to that of sample **1**, shows that an arrangement of N-terminal regions (residue 1-16) of peptides different from that in soluble oligomers is achieved in protofibrils. It is unlikely that the absence of DEER time trace is due to the collapse of copper spins in the fibrils. Copper spin silencing because of the formation of a *S* = 0 spin state does not occur even in the prion N-terminus, where the Cu–Cu distance approaches 3.5 Å.^71^ The absence of a detectable dipolar coupling between Cu ions can be either due to a Cu–Cu distance shorter than 1 nm, where DEER technique is not sensitive, or to a broad distribution of Cu–Cu distances, with a few contributions of distances in the 2–8 nm range. A low chance for a regular positioning of N-termini in the fibril is shown by the structural disorder affecting N-termini observed in most of the available experimental studies. The DEER trace observed for sample **1**, and not observed in sample **2**, demonstrates that regular positioning of N-termini is favored in small toxic oligomers.

We finally performed the DEER measurement for sample **1** incubated for 72 h. In the conditions of sample **1** (25 μM concentration and quiescent incubation at 25 °C) Aβ(1-42) fibrils never form, even at long incubation times:^46^ The aggregation pathway is definitely diverted from fibrils to amorphous particles. This final state of sample **1**, termed A–, is also less toxic than early soluble species (termed A+). No DEER signal is present for this sample. This shows that, in any of the late stages and less toxic forms of aggregation, the N-terminal chains do not form regular registers. The regular Cu–Cu distance displayed by the DEER measurement after 24 h is a fingerprint of early and more toxic species on the pathway toward aggregated forms. After we consider that the ESR spectra at pH 7.7–7.8 are quite insensitive to the incubation time up to 48 h (Figure 2), a change of Cu coordination after 72 h of incubation is likely to be excluded.

The change in structure of N- and C-termini in protein oligomers has been correlated to toxicity in the well-studied HypF-N protein.^72^ The type A toxic oligomers of HypF-N protein contain N-terminal regions that are less disordered than in nontoxic type B oligomers. The difference in N-termini structure has the consequence of exposing hydrophobic residues of the C-termini to the solvent in toxic species, while nontoxic species have more chances to settle C-termini into stable aggregates. A similar transition between closed fibrillar aggregates and globular or open forms of Aβ(1-40) has been hypothesized on the basis of ion mobility mass spectrometry (IM-MS) data.^43,73^

More generally, the effect of N-termini on the aggregation propensity of Aβ and on interactions with lipid membranes has been investigated because of the polar and hydrophilic nature of region 1-16.^74,75^ Indeed, the modulation of N-terminal charge by metal ion binding has been proposed as a key effect in cell toxicity.^76^ The N-terminal charge and its screening because of binding cations like Cu(II) is also the basis of the relevance of N-truncated forms in Aβ aggregation and toxicity.^77^

Due to the lack of experimental structural information concerning Aβ N-termini and to the potential interest of such domains, many computational studies have been performed to provide such missing information. The distribution of distance between metal ions bound to Aβ peptides has been investigated, for instance, in ref (78), where Zn-Aβ(1-42) was modeled in 12 different fibril states. The binding of Zn was different than that assumed for Cu in this work; yet, the metal ion binding was confined to the N-terminus in all models. In all of the different arrangements reported, regular Zn–Zn distances can be estimated in the 2–4 nm range when the parallel arrangement of C-termini^79^ was used. In these cases, Zn-cross-linked dimers also become possible. A similar result was obtained for Cu–Aβ(1-40) models,^80^ where regular registers of Cu–Cu distance were obtained by using the parallel arrangement of C-termini.

It must be noticed that all of the structures in the Protein Data Bank (PDB) of Aβ(1-42) fibrils^79,81−84^ display monomers assembled into β sheets with interpeptide hydrogen bonds mostly oriented along a direction perpendicular to the *z* (long) axis of the fibril. As for this arrangement, the peptides are parallel to each other. The assembly formed by each parallel arrangement is associated with one or more assemblies forming a fiber. The mutual orientation of peptides in the *xy* plane of the fiber is always antiparallel in the interface region. The transition between antiparallel and parallel arrangement in the *xy* plane of the fiber has been particularly investigated, because the parallel *xy* orientation favors the antiparallel arrangement along the *z* axis, thus destabilizing the fibril. For instance, the Iowa D23N mutation induces a fibril destabilization and a higher toxicity of Aβ(1-42) peptides.^85,86^

By combining available modeling studies with the structural information in the PDB, we can argue that, in fibrils, there is a chance for an organization of pairs of N-termini induced by metal ion binding, but this chance is limited to separated pairs of tails. This limitation is due to the strong interactions between pairs of C-termini, which can form long registers as β sheets, while N-termini act, even when involved in metal-bridged dimers, as separated entities. On the contrary, oligomers have a larger chance for a structural organization of N-termini, with the consequence of hindering a stable assembly of C-termini.

We remark that oligomer morphology does not change with Cu addition, as shown by AFM images. The relevance of the observation of a dominant Cu–Cu dipolar coupling in the observed oligomers is broader than the experimental support for Cu–Aβ(1-42) oligomers formed by dimers with a defined Cu-cross-linked topology. The constraint concerning peptide N-termini arrangement can be relevant for Cu-free peptides as well. Therefore, these ESR experiments and the structures consistent with them add an important piece of information to the relative position of peptide termini in toxic oligomers in a physiological environment. This information has rarely been obtained by other experimental techniques, and in those reported cases, like cryo-EM or ssNMR results, effects of solid state packing may affect the results.^84^

The preparation of the samples is a crucial step of this work. The established procedure used for preparing the cytotoxic oligomers commonly indicated as amyloid derived diffusible ligands (ADDLs), which resemble those found in the brain of AD patients,^87^ could not be adopted here. In fact, the F12 medium used in that preparation contains many amino acids and anions that sequester copper ions from Aβ binding, with the result that copper cannot be used as a probe for oligomer structure.

## Conclusions

We performed DEER experiments on Cu–Aβ(1-42) samples in toxic oligomeric state (A+), in protofibrillar state, and in low-toxicity amorphous state (A−). The addition of the Cu^2+^ magnetic probe was performed with no change in oligomers’ morphologies with respect to Cu-free samples in the same conditions, as monitored by atomic force microscopy.

We observed oscillations in DEER time trace only for the A+ state. By comparing the Cu–Cu distance distribution obtained by analyzing the DEER time trace with atomistic models of Cu–Aβ(1-42) tetramers, we demonstrate that assembly of Cu-cross-linked dimers into globular tetramers well-explains the DEER experiment.

The approach of Aβ(1-42) N-termini monitored by Cu–Cu distance shows that interactions between N-termini in A+ toxic state disfavor the parallel assembly of C-termini typical of most fibril structures. Therefore, we confirm the hypothesis that N-termini assembly induces the stabilization of toxic oligomers that are off-pathway to protofibrils, similar to other proteins that aggregate into fibrils.

## Materials and Methods

### Sample Preparation and pH Measurement

We prepared two samples displaying different morphology: sample **1**, constituted by cytotoxic oligomers,^45^ indicated as A+ or A-, depending on incubation time; sample **2**, constituted by protofibrils.^46^ The preparation is the same as of Cu-free samples except for the introduction of Cu^2+^. Briefly, Aβ(1–42) monomers were prepared from the commercial peptide powder (Cayman Chemical, USA). The powder was dissolved in 100% hexafluoroisopropanol (HFiP, Sigma-Aldrich, Saint Louis, MO, USA) and stock solutions were stored at −20 °C. Before preparing the oligomers, the solvent was evaporated and Aβ(1–42) was dissolved in 50 mM NaOH reaching a peptide concentration of 1 mg/mL, corresponding to 221 μM. The solution was sonicated for 30 s. A solution of copper acetate and PBS characterized by a copper concentration of 221 μM was prepared. This copper solution was added to an equal volume of that containing the peptide, and PBS was added up to achieving the desired final concentration. All additions were performed by gently mixing volumes. The dilution was such that the resulting concentration of Aβ(1-42) was 25 and 75 μM in the case of samples **1** and **2**, respectively. Ultrapure water was used. The preparation was centrifuged at 22 000 *g* for 30 min to remove possible small insoluble particles, and the supernatant solution was incubated at 25 °C under quiescent conditions.^45^

The pH of sample **1** was measured after incubation times of 0, 24, and 48 h. In addition, in order to monitor copper coordination as a function of pH by ESR, the pH of the original samples, incubated for different times, was adjusted to values ranging between 5 and 10 by the addition of small aliquots of H_2_SO_4_ or NaOH aqueous solutions. Specifically, the pH was set to 6 values in the order 10, 9, 8, 7, 6, 5, which are the values investigated in previous reports about Aβ(1-16) and Aβ(1-28).^55^ After the pH measurement in the original 5 mL sample at a given incubation time, 200 μL was extracted for ESR measurement. The extracted aliquot was added to glycerol (10% volume addition), inserted in the ESR tube, and frozen in liquid nitrogen. The stirred solution was then added to 11 μL of NaOH (0.5 M) to obtain pH = 10. The solution was stirred for a few minutes before a second 200 μL extraction of the sample for the ESR measurement was done, as in the previous stage. The stirred solution was then added to 26 μL of sulfuric acid (0.5%) to obtain pH = 9. Extraction and pH shift were repeated to obtain samples at the different pH values. Added volumes of sulfuric acid changed to a maximal value of 90 μL when in the buffer region of PBS. The peptide concentration for the lowest pH = 5 was 23.5 μM, and the volume was 3.9 mL. This procedure was repeated for sample **1** at the three different incubation times. At each incubation time, the time elapsed since the first pH measurement until the last insertion into ESR tube was shorter than 1 h.

The pH was measured at 21 °C with a Fisher Scientific accumet (TM) AE150 equipment connected to a pH electrode (single junction and epoxy body VWR electrode) according to a well-tested protocol.^88,89^ The system was calibrated before every measurement with two standard pH buffers at pH 4.01 and 7.01 (HI 7004, HI 7007).

For all sample preparations Ultrapure Milli-Q water was used, together with glycerol (99.5%, Aldrich), copper acetate (anydrous, 99.99%, Aldrich), NaOH (98%, Aldrich), and H_2_SO_4_ (95–97%, Fluka). All compounds were used as supplied. Copper acetate was kept in the stove before the weighting.

### Atomic Force Microscopy

Morphology and size of samples **1** and **2** were inspected by tapping mode AFM, according to previous works.^46,90,91^ A 10 μL volume of the sample was dried on top of freshly cleaved mica substrates at room temperature for 1 h, followed by rinsing in Milli-Q water to remove salts and drying under a gentle nitrogen flow. PBS solution was also measured as a control experiment. Samples were immediately imaged using a JPK NanoWizard III Sense (Berlin, Germany) scanning probe microscope operating in AFM mode (maximum z-scan size 15 μm). Single-beam uncoated silicon cantilevers (μMash HQ:NSC15 Cr–Au BS) were used. Drive frequency was between 250 and 300 kHz, and the scan rate was 0.5 Hz.

### Electron Spin Resonance

The ESR measurements were conducted on a Bruker ELEXSYS E580 spectrometer provided by the INSTRUCT-ERIC EU infrastructure. All samples were rapidly frozen after addition of glycerol at 10% in volume (1 volume of glycerol mixed to 10 volumes of the sample).

X-band (9.4 GHz) continuous-wave (CW) ESR spectra were collected using the ELEXSYS Super High Sensitivity Probehead (ER 4122SHQE) resonator, equipped with an Oxford helium temperature regulation unit, at the temperature of 20 K. The parameters used were as follows: The microwave power was 0.2 mW, and the magnetic field modulation amplitude was 10 G; the modulation frequency was 100 kHz, and the receiver gain was 60 dB. The spectra were accumulated 16 times to increase the signal-to-noise ratio.

The DEER experiment, used to measure distances between the unpaired electron spins of Cu^2+^ ions, was conducted on the same spectrometer at Q-band using the standard EN 5107D2 resonator. The system was equipped with an Oxford helium temperature regulation unit, and the data were acquired at 15 K at a repetition rate of 1 kHz.

The well-described 4-pulse DEER sequence was applied.^92^ The scheme consists of π/2 – τ_1_ – π – τ_1_ + τ_2_ – π – τ_2_ applied at the observer frequency. In addition, a pump pulse of flip angle π at the pump frequency is applied at a variable delay time *t* between the second pulse of the observer sequence and the pump pulse. The lengths of the π and π/2 pulses of the observer sequence were 64 and 32 ns, respectively, whereas the length of the pump pulse was 60 ns. The interpulse delay τ_1_ was 200 ns, and the τ_2_ was set according to the spin–spin relaxation time of the sample. Initial *t* of 100 ns was set with an increment step of 10 ns and a total number of increments of 200. The field positions of pump and detection pulses, located at the Cu^2+^ spectrum maximum in the *g*_*y*_ region, were separated by 25 G. The total acquisition time was 5–6 h. The measurements were performed with an eight-step nuclear modulation average.

The DeerAnalysis2013 open-source software,^93^ available for MatLab,^94^ was used for processing and analyzing DEER data. Background echo decay was corrected by using a homogeneous three-dimensional spin distribution. Tikhonov regularization^60^ was applied to the corrected dipolar evolution data set to obtain interspin distance distributions (see also discussion about orientational selectivity in the Results and Discussion section).

### Molecular Statistics

We analyze experimental data using tetramer structures obtained as described in our previous works.^42,63,95^ The sampling, based on computational empirical models, is summarized below, as for Cu–Aβ(1-42) tetramers of stoichiometry 1:1 and 1:2.

We used two models of Cu–Aβ binding. In the first model, each Cu ion is bound to a single peptide via N and O of Asp 1, Nδ of His 6, and Nϵ of His 13. This will be indicated as non-Cu-cross-linked model (Cu1), hereafter. In the second model, each Cu ion is bound to Asp 1 and His 6 of one peptide (say A) and His 13 of the other peptide (B), via the same ligand atoms in each residue. This will be indicated as Cu-cross-linked model (Cub), hereafter. A further model uses the same Cu-binding model of Cu1 but in a 1:2 Cu–Aβ ratio. In this model, Cu is bound to monomer A and forms a dimer with Cu-free monomer B. This will be indicated as model Cuh.

**Figure 7:**
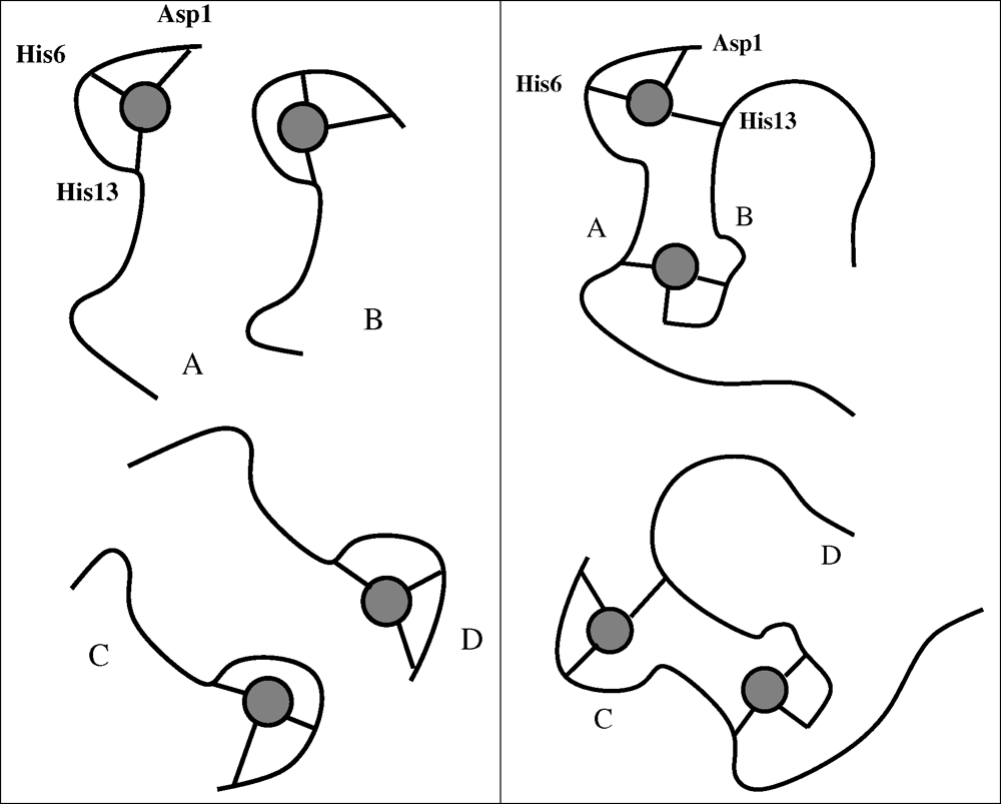
Schematic representations of computational models of tetramers used in this work: The circle represents Cu ion; the curves represent Aβ(1-42) peptides. (a) Cu1 model, with each Cu ion bound to each peptide. (b) Cub model, with Cu ions forming cross-links between the monomers in each preformed dimer. Top and bottom dimers represent the preformed AB and CD dimers that approach each other to form the ABCD tetramer.

Tetramers are built as dimers of dimers (AB and CD). A schematic picture of the type of models used in this work is displayed in Figure 7. In model Cu1, each dimer is composed by monomers, with each peptide bound to a Cu ion and no cross-links connecting the monomers. In model Cub each dimer contains two Cu-cross-links connecting the two monomer peptides. In model Cuh (not displayed), each dimer contains only one Cu ion bound as in Cu1. Monomers A and C are, therefore, bound to Cu ions as in Cu1, and Cu-free peptides (B and D) are intercalated within the Cu-bound monomers (A and C). Summarizing, tetramer models are indicated as Cu1 (4 × Cu–Aβ(1-42), Figure 7a), Cub (2 × [Cu–Aβ(1-42)]_2_, Figure 7b), and Cuh (2 × [Cu–Aβ(1-42)–Aβ(1-42)], not displayed), respectively. The Cu binding to Aβ(1-42) in Cu1, Cub, and Cuh tetramers is that consistent with the dominant species identified by interpretation of ESR spectra at pH ∼ 7.^64−66^ Even though slight differences occur between the cited studies in terms of the ligand atoms, the models include the experimental constraint of Asp 1 and His 6 and one of the two His in the 13–14 pair coordinated to Cu^2+^.

Initial configurations of Cu–Aβ(1-42) monomers, [Cu–Aβ(1-42)]_2_ Cu-cross-linked dimers, and [Cu–Aβ(1-42)–Aβ(1-42) dimers were selected as the most representative structures obtained with 1 μs atomistic molecular dynamics (MD) simulations performed in explicit water solvent.^95^ The selection of initial configurations was performed according to the maximally populated peak in the gyration radius (*R*_g_) and solvent accessible surface area (SASA) probability map in all cases. The force field was Amber 99SB,^96^ integrated with the parametrization of the chosen Cu-binding site.^95^

Indicating the monomers with letters A–D, we built different assemblies of A and B monomers into dimers and built assemblies of AB and CD dimers into tetramers. This was done by placing two particles in space, monomers and dimers, for building dimers and tetramers, respectively. We placed the particles with the selected structures and with random orientations with centers of mass at an approximate distance of 2 nm. The particles were inserted into orthorhombic simulation cells filled of water molecules described as in the TIP3P model^97^ and a neutralizing amount of NaCl 0.1 M.

We performed MD simulations with time-step of 2 fs and rigid constraints for bonds involving hydrogen atoms, in the NPT statistical ensemble of 128 initial mutual orientations of the particles. Pressure was 1 bar and temperature was 300 K. We used the multiple-walkers metadynamics to separate the independent trajectories, one with respect to each other. The diversity among different walkers is limited, here, to the mutual orientation of the peptide that forms each assembly: monomers, when dimers are built, and dimers, when tetramers are built. We performed a single multiple-walkers simulation for 128 replica of the system. The spreading of walkers among independent trajectories was performed by adding a bias potential constructed according to the altruistic method,^98^ with a collective variable chosen as the number of salt bridges within each monomer. This choice was dictated by the observation that this variable is particularly effective in changing the peptide structure,^95^ thus allowing a wider sampling of different structures within the multiple walkers. After MD simulation of 20 ns in the presence of the progressively built (history-dependent) bias, 2 ns were performed with no bias. The last 1 ns was used for averaging, using one configuration every 10 ps of simulation (100 configurations per walker). The NAMD 2.10 package^99^ was used for the simulations, with most of the MD simulation parameters chosen according to standard procedures. Because some walkers were unstable, the total number of collected configurations for Cu1 was 126 × 100 (2 walkers ignored) and 127 × 100 (1 walker ignored), for Cub and Cuh, respectively.

As in previous published analysis, tetramers are defined when, in the collected statistics, the ratio between the total solvent accessible surface area (SASA(ABCD)) divided by the sum of the SASA of the two constituent dimers (SASA(AB) + SASA(CD)) is lower than 0.95. Tetramers were 5650 over 12 600 (45%), 3037 over 12 700 (24%), and 4792 over 12 700 (38%) for Cu1, Cub, and Cuh, respectively.

The selection of representative structures among the tetramers was performed by finding individuals that are contained in peaks of maximal population of distributions of different structural quantities. Here, we used the Cu–Cu distance within preformed dimers (AB) and the radius of gyration, *R*_g_.
